# Tumor suppressive function of Matrin 3 in the basal-like breast cancer

**DOI:** 10.1186/s40659-020-00310-6

**Published:** 2020-09-25

**Authors:** Jaehyuk Yang, Seung Jun Lee, Yongseok Kwon, Li Ma, Jongchan Kim

**Affiliations:** 1 Department of Life Sciences, Sogang University, 35 Baekbeom-ro, Mapo-gu, Seoul, Republic of Korea; 2 Department of Chemistry, Sogang University, Seoul, Republic of Korea; 3 Department of Experimental Radiation Oncology, University of Texas MD Anderson Cancer Center, Houston, TX USA

**Keywords:** MATR3, Basal-like breast cancer, Triple-negative breast cancer, Apoptosis, Tumor suppressor, Epithelial-mesenchymal transition, YAP/TAZ, Biomarker

## Abstract

**Background:**

Basal-like breast cancer (BLBC) or triple-negative breast cancer (TNBC) is an aggressive and highly metastatic subtype of human breast cancer. The present study aimed to elucidate the potential tumor-suppressive function of MATR3, an abundant nuclear protein, in BLBC/TNBC, whose cancer-relevance has not been characterized.

**Methods:**

We analyzed in vitro tumorigenecity by cell proliferation and soft agar colony formation assays, apoptotic cell death by flow cytometry and Poly (ADP-ribose) polymerase (PARP) cleavage, epithelial-mesenchymal transition (EMT) by checking specific EMT markers with real-time quantitative PCR and in vitro migration and invasion by Boyden Chamber assays. To elucidate the underlying mechanism by which MATR3 functions as a tumor suppressor, we performed Tandem affinity purification followed by mass spectrometry (TAP-MS) and pathway analysis. We also scrutinized *MATR3* expression levels in the different subtypes of human breast cancer and the correlation between *MATR3* expression and patient survival by bioinformatic analyses of publicly available transcriptome datasets.

**Results:**

MATR3 suppressed in vitro tumorigenecity, promoted apoptotic cell death and inhibited EMT, migration, and invasion in BLBC/TNBC cells. Various proteins regulating apoptosis were identified as MATR3-binding proteins, and YAP/TAZ pathway was suppressed by MATR3. *MATR3* expression was inversely correlated with the aggressive and metastatic nature of breast cancer. Moreover, high expression levels of *MATR3* were associated with a good prognosis of breast cancer patients.

**Conclusions:**

Our data demonstrate that MATR3 functions as a putative tumor suppressor in BLBC/TNBC cells. Also, MATR3 potentially plays a role as a biomarker in predicting chemotherapy-sensitivity and patient survival in breast cancer patients.

## Background

Basal-like breast cancer (BLBC) or triple-negative breast cancer (TNBC) is characterized by a lack of hormone receptors (estrogen receptor and progesterone receptor) and high expression or amplification of human epidermal growth factor receptor 2 (*HER2*) [1, 2]. Because of the nature of BLBC/TNBC, this breast cancer subtype does not respond to the targeted endocrinal hormone therapies such as tamoxifen and aromatase inhibitors which are proven to be highly effective [3, 4]. Moreover, BLBC is clinically aggressive and its pathogenesis is poorly understood, so it is a huge challenge, compared to other breast cancer subtypes such as hormone receptor-positive and HER2-positive tumors.

Matrin 3 (MATR3) is an abundant nuclear protein with DNA- and RNA-binding domains [5, 6]. Missense mutations in *MATR3* including S85C, F115C, P154S, and T622A have been associated with a rare human disorder called Amyotrophic lateral sclerosis (ALS) [7]. MATR3 has been shown to play roles in mRNA stabilization, nuclear retention of hyper-edited RNAs [8, 9], and RNA splicing [10]. In cancer, *MATR3* was identified as one of the genes deleted from the chromosome 5 of human BLBC (primary, metastasized, and xenografted in immune-deficient mice) along with *CTNNA1, LRRTM2,* and *SNORA74A* [11]. Among them, *CTNNA1,* encoding α-catenin, has been shown to have tumor-suppressive functions in E-cadherin-negative BLBC [12], which suggests that *MATR3* may also function as a tumor suppressor gene but its role in cancer remains elusive.

In the present study, we aimed to investigate the role of MATR3 in breast cancer, especially in BLBC. We found that MATR3 controls tumorigenecity, apoptotic cell death, migration, and invasion by regulating epithelial-mesenchymal transition (EMT) in BLBC cells. Besides, we examined MATR3-interacting proteins by Tandem affinity purification followed by mass spectrometry (TAP-MS) to identify apoptosis-controlling proteins, and enriched pathway to find a signaling pathway that is the most significantly regulated by MATR3. Then, we elucidated its clinical correlation in human breast cancer by scrutinizing bioinformatic dataset analyses.

## Results

**Fig. 1:**
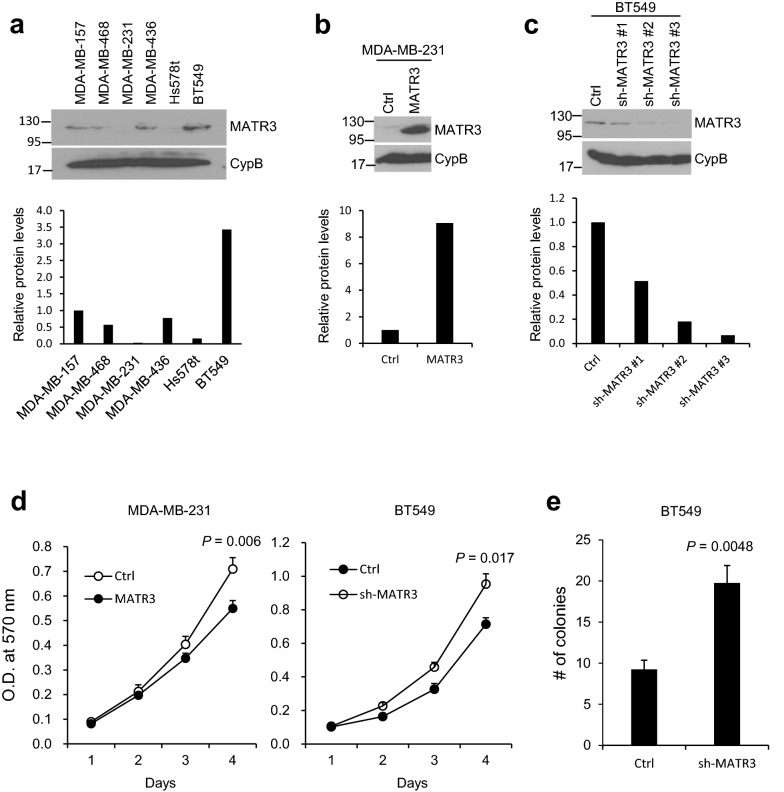
Comparison of MATR3 protein levels in a panel of basal-like/triple-negative human breast cancer cell lines. **a** Endogenous MATR3 protein levels were examined in a small panel of basal-like/triple-negative breast cancer cell lines by western blotting. The lower panel demonstrates the relative normalized MATR3 protein levels. **b** MATR3 was transiently overexpressed in MDA-MB-231 cells and the expression was determined by western blotting. The lower panel demonstrates the relative normalized MATR3 protein levels. **c** MATR3 was silenced by shRNAs and the expression was determined by western blotting. The lower panel demonstrates the relative normalized MATR3 protein levels. **d** *In vitro* cell proliferation was compared between control and MATR3-overexpressing MDA-MB-231 and between control and MATR3-depleted BT549 cells. *n* = 4 per group. **e** Anchorage-independent growth was examined to compare in vitro tumorigenesis by soft agar colony formation assay. *n* = 4 per group. Statistical significance in **d** and **e** was determined by an unpaired *t*-test. Error bars are s.e.m

To compare the relative MATR3 expression levels in a small panel of human breast cancer cell lines with basal-like/triple-negative breast cancer (BLBC/TNBC) properties, we performed western blotting with a specific antibody against MATR3. As shown in Fig. 1a, BT549 cells express the most, and MDA-MB-231 cells express the least protein levels of MATR3. Thus, we overexpressed and silenced MATR3 from MDA-MB-231 and BT549 cells, respectively (Fig. 1b, c). Among three short hairpin RNAs (shRNAs) against *MATR3*, clone #3 achieved the best gene silencing (~ 93%), so we used this clone in the subsequent assays. Using the cell lines, we examined whether MATR3 levels influence the basal cell proliferation, and found that MATR3 overexpression inhibited and its depletion increased in vitro cell proliferation in MDA-MB-231 and BT549 cells, respectively (Fig. 1d). Also, MATR3 depletion significantly promoted colony formation in the soft agar assays (Fig. 1e), which suggests that MATR3 is necessary to suppress tumorigenecity in the BLBC/TNBC cells.

**Fig. 2:**
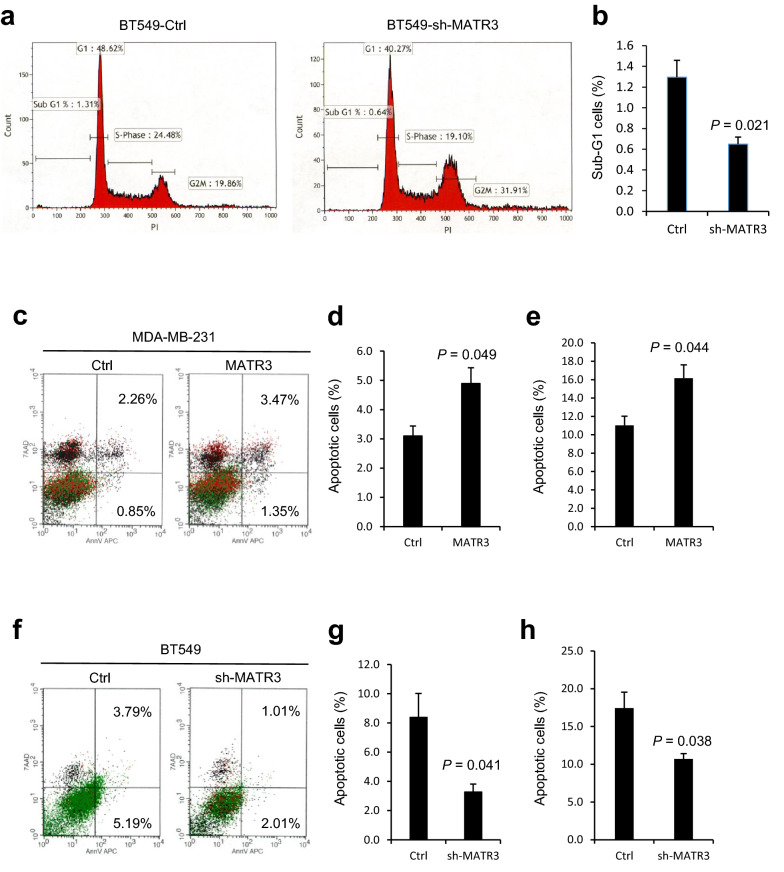
MATR3 promotes apoptotic cell death gauged by flow cytometry. **a** Cell cycle was analyzed in BT549 cells introduced with non-silencing control or short hairpin RNA against MATR3 (sh-MATR3) using propidium iodide by flow cytometry. **b** Sub-G1 cells were quantitated and compared between the BT549 cells introduced with non-silencing control and sh-MATR3. MDA-MB-231 (**c**, introduced with empty vector and MATR3-overexpression vector) and BT549 cells (**f**, introduced with non-silencing control and sh-MATR3) were cultured in the serum-free and growth factor-free medium for 24 h and subjected to flow cytometry following Annexin V and 7-AAD staining. (**d** and **g**) (Early, Annexin V +/7-AAD-, and late, Annexin V +/7-AAD +) apoptotic cells from **c** and **f** were quantitated and compared. (**e** and **h**) MDA-MB-231 (**e** introduced with empty vector and MATR3-overexpression vector) and BT549 cells (**h** introduced with non-silencing control and sh-MATR3) were treated with 0.5 μM Staurosporine for 24 h and subjected to flow cytometry following Annexin V and 7-AAD staining. Then (early and late) apoptotic cells were quantitated and compared. *n* = 3 per group. Statistical significance in **b, d, e, g,** and **h** was determined by an unpaired *t*-test. Error bars are s.e.m

Next, we questioned whether MATR3 suppresses tumorigenecity by regulating the cell cycle. Therefore, we performed flow cytometric analysis by staining cellular DNA with propidium iodide. Compared to the control cells, MATR3-depleted BT549 cells displayed slightly but significantly less sub-G1 cells (1.30% → 0.65%), which suggests that MATR3 controls cell death (Fig. 2a, b). To examine whether MATR3 regulates apoptotic cell death, we stained the cells with Annextin V and 7-AAD. When MATR3 was overexpressed in MDA-MB-231 cells, (early and late) apoptotic cells were significantly increased from 3.1% to 4.9% (Fig. 2c, d). To validate this change, we challenged the cells with Staurosporine (STS), a well-known apoptosis-inducer [13], and tested whether MATR3 promotes apoptotic cell death. STS increased apoptosis in control cells (11.0%) and MATR3-overexpression further increased apoptosis in MDA-MB-231 cells (16.2%) (Fig. 2e). When MATR3 was abrogated by shRNA in BT549 cells, however, apoptosis was suppressed from 8.4% to 3.3% (Fig. 2f, g), compared to the apoptosis in the control cells. When STS was treated, apoptosis in control BT549 cells was 17.4% and MATR3 silencing suppressed it to 10.7% (Fig. 2h).

**Fig. 3:**
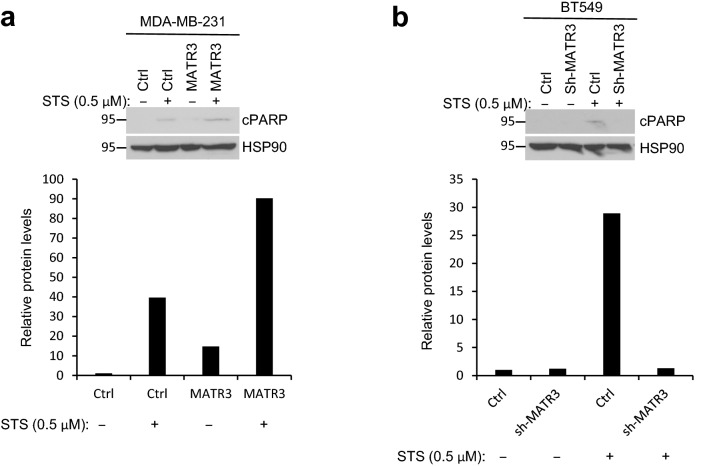
MATR3 promotes apoptotic cell death gauged by PARP cleavage. MDA-MB-231 (**a** introduced with empty vector or MATR3-overexpression vector) and BT549 cells (**b** introduced with non-silencing control or sh-MATR3) were treated with 0.5% DMSO (−) or 0.5 μM Staurosporine (+) for 24 h and subjected to western blotting. Cleaved PARP (cPARP) was detected with a specific antibody. The lower panels demonstrate the quantitation of the relative normalized cPARP protein levels

Poly (ADP-ribose) polymerase (PARP) is a nuclear enzyme that plays a role in DNA damage repair [14, 15]. When the cells undergo apoptosis, caspases are activated to cleave several key proteins including PARP [16], yielding an ~ 85 kDa fragment [17]. Therefore, we treated Staurosporine to two basal-like breast cancer cell lines and examined whether MATR3 further controls apoptosis by affecting PARP cleavage. In MDA-MB-231 cells, upon Staurosporine treatment, MATR3 overexpression increased apoptosis, as gauged by 2.27-fold more PARP cleavage than the control cells (Fig. 3a). In BT549 cells, MATR3 silencing reduced apoptosis, as determined by 21.83-fold less PARP cleavage upon Staurosporine treatment (Fig. 3b). PARP cleavage upon Staurosporine confirms that MATR3 further promotes and its depletion suppresses apoptotic cell death in basal-like breast cancer cells. In addition, we attempted to find interacting proteins with MATR3 that play a role in cellular apoptosis. Using Tandem affinity purification followed by mass spectrometry (TAP-MS), we identified multiple numbers of proteins involved in apoptotic cell death as shown in Table 1. Taken together, flow cytometric analyses, PARP cleavage and the TAP-MS demonstrate that MATR3 promotes and its depletion suppresses apoptotic cell death in basal-like breast cancer cells potentially by interacting with apoptosis-controlling proteins.

**Table 1:** List of MATR3-interacting proteins identified by TAP-MS analysis

Peptide Hits	Protein	PMID
191	MATR3	
28	DBC1	18235502
27	LARP1	20430826
10	UPF1	30719122
7	FASN	16740734
5	PAICS	30097015
4	EWSR1	30481590
4	USP10	31301769
4	NCKAP1	10673335
4	QKI	32047543
3	NUDT21	30349365
3	SFPQ	29719248
3	CPSF1	32046510
3	NONO	29620226
2	CFL1	31990066
2	GEMIN5	17541429
2	TAF15	23128393
2	OLA1	32045367
1	BRE	28333135
1	CHEK1	12554671
1	PDK1	11102805

MATR3-interacting proteins known to regulate apoptotic cell death are listed. PubMed IDs (PMID) of related literature demonstrating the regulation of apoptotic cell death are shown

**Fig. 4:**
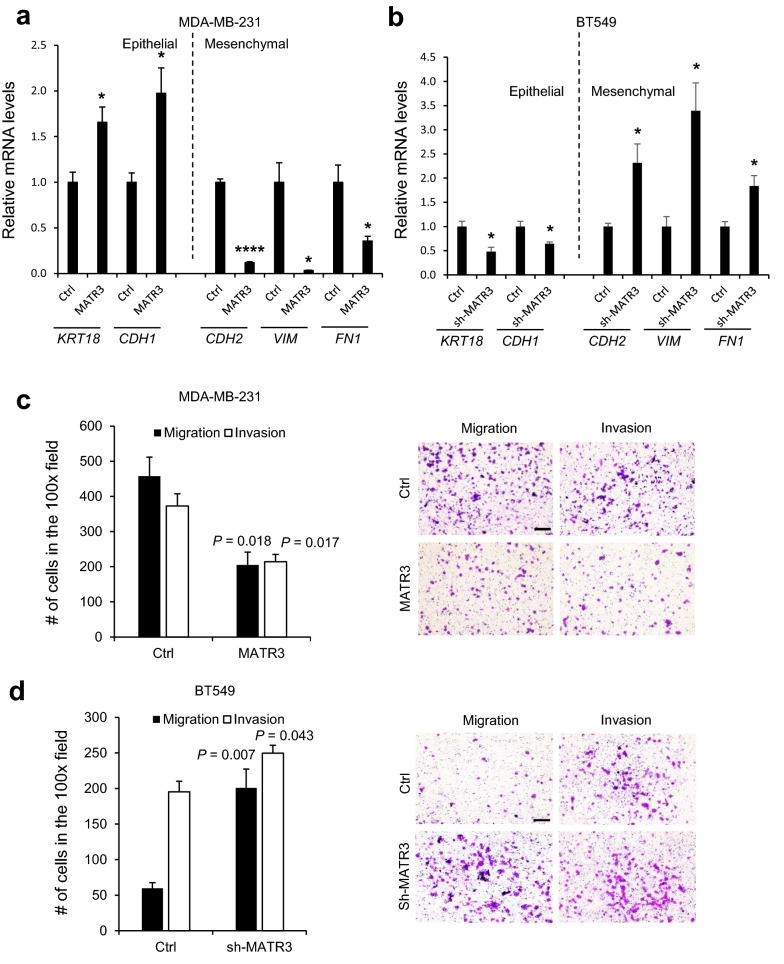
MATR3 suppresses EMT (epithelial-mesenchymal transition), migration, and invasion. **a** Real-time quantitative PCR was performed to measure relative mRNA levels of epithelial (Cytokeratin 18 and E-cadherin) and mesenchymal (N-cadherin, Vimentin and Fibronectin) markers in MDA-MB-231 (**a** introduced with empty vector and MATR3-overexpression vector) and BT549 cells (**b** introduced with non-silencing control and sh-MATR3). *In vitro* 3D migration and invasion of MDA-MB-231 (**c** introduced with empty vector and MATR3-overexpression vector) and BT549 (**d** introduced with non-silencing control and sh-MATR3) were examined using Boyden Chambers. Right panels in **c** and **d** show representative images of migration and invasion. Scale bars, 200 μm. *n* = 3 per group. Statistical significance in **a–d** was determined by an unpaired *t*-test. Error bars are s.e.m. * and **** indicate *P* < 0.05 and *P* < 0.0001, respectively

We then explored whether MATR3 affects migration and invasion which decide the metastatic nature of the cancer cells. To examine whether MATR3 regulates epithelial-mesenchymal transition (EMT), we performed RT-qPCR for a few genes related to EMT. MATR3 overexpression in MDA-MB-231 cells induced epithelial markers such as *KRT18* (Cytokeratin 18) and *CDH1* (E-cadherin) and reduced mesenchymal markers such as *CDH2* (N-cadherin), *VIM* (Vimentin) and *FN1* (Fibronectin) (Fig. 4a). On the contrary, MATR3 depletion in BT549 cells suppressed epithelial markers and increased mesenchymal markers (Fig. 4b). To investigate whether MATR3 functionally regulates migration and invasion, we performed in vitro migration and invasion assays using Boyden Chambers. MATR3 overexpression in MDA-MB-231 significantly suppressed cell migration and invasion (Fig. 4c), which were promoted in BT549 cells when MATR3 expression was abrogated (Fig. 4d). The results implicate that MATR3 suppresses cell migration and invasion by affecting EMT.

**Fig. 5:**
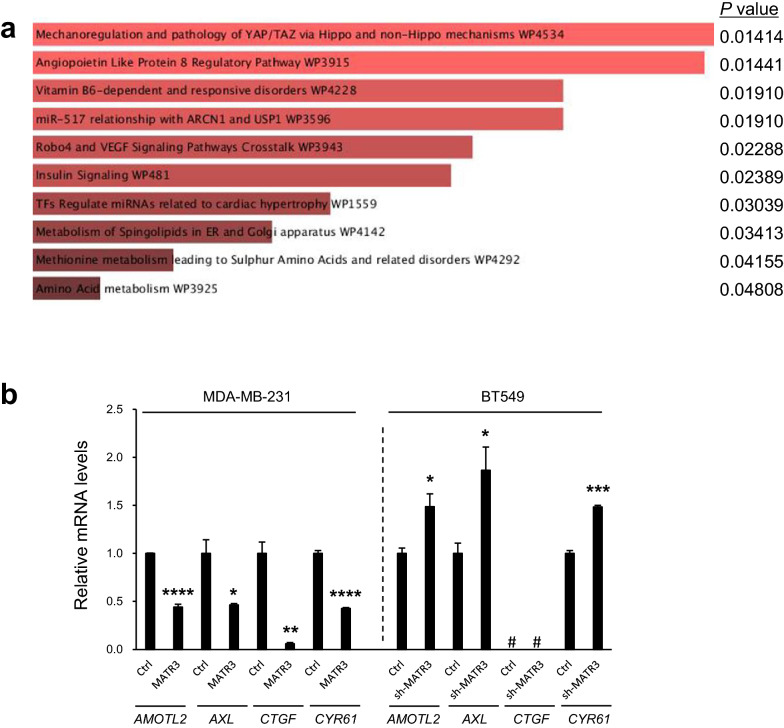
MATR3 suppresses YAP/TAZ pathway. **a** YAP/TAZ pathway was the most significantly modulated pathway by MATR3. Enriched pathways are listed according to the statistical significance. **b** RT-qPCR was performed to compare the expression of four YAP/TAZ target genes; between control and MATR3-overexpressing MDA-MB-231 cells, and between control and MATR3-depleted BT549 cells. *n* = 3 per group. Statistical significance in **a** and **b** was determined by pre-set analytical methods of the online tool, and an unpaired *t*-test, respectively. Error bars are s.e.m. *, **, *** and **** indicate *P* < 0.05, *P* < 0.01, *P* < 0.001 and *P* < 0.0001, respectively. #: not detectable quantity

To further elucidate the underlying mechanism by which MATR3 functions as a putative tumor suppressor, we explored the signaling pathway altered by MATR3. To do so, we obtained microarray results consisting of the genesets differentially expressed in MATR3-silenced cells [9] and analyzed the enriched signaling pathway using Enrichr, an online bioinformatics tool [18]. As shown in Fig. 5a, YAP/TAZ pathway was the most significantly (*P* = 0.014) altered by MATR3. YAP and TAZ are oncogenic transcriptional coactivators that suppress apoptosis and induce EMT [19–21] through associating with TEAD transcription factors, promoting tumorigenesis and metastasis. Therefore, we examined whether the expression of their transcriptional target genes, *AMOTL2, AXL, CTGF*, and *CYR61* [22, 23] is regulated by MATR3. RT-qPCR analysis revealed that the expression of the target genes was significantly reduced in MATR3-overexpressing MDA-MB-231 cells and increased in MATR3-silenced BT549 cells (Fig. 5b) compared to their control cells, which suggests that MATR3 promotes apoptosis and suppresses EMT, migration, and invasion, possibly by inhibiting YAP/TAZ pathway.

**Fig. 6:**
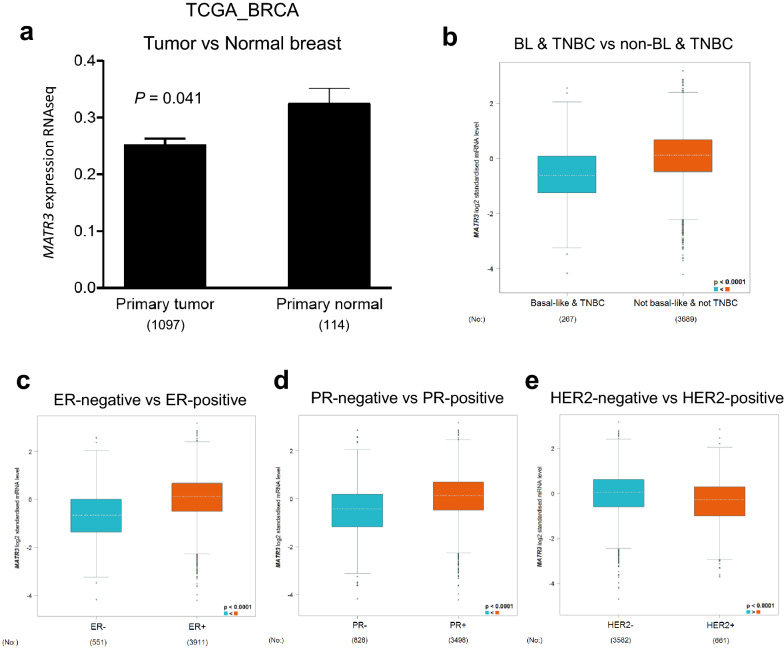
*MATR3* expression is inversely correlated with the aggressiveness of breast tumors. **a** RNA-sequencing datasets from TCGA were analyzed and *MATR3* mRNA levels were compared between primary breast tumors and normal breast tissues. *n* = 1097 (primary breast tumors) and 114 (normal breast tissues). **b** RNA-sequencing datasets at bc-GenExMiner were analyzed to compare *MATR3* mRNA levels between basal-like (BL) and triple-negative breast cancer (TNBC) and not-BL and not-TNBC. *n* = 267 (BL & TNBC) and 3689 (not-BL & not-TNBC). RNA-sequencing datasets at bc-GenExMiner were analyzed to compare *MATR3* mRNA levels between hormone receptor (**c**, estrogen receptor; **d**, progesterone receptor)-negative and -positive breast cancers. *n* = 551 (ER-negative), 3911 (ER-positive), 828 (PR-negative) and 3498 (PR-positive). **e** RNA-sequencing datasets at bc-GenExMiner were analyzed to compare *MATR3* mRNA levels between human epidermal growth factor receptor 2 (*HER2*)-negative and -positive breast cancers. *n* = 3582 (*HER2*-negative) and 661 (*HER2*-positive). Statistical significance in **a** was determined by an unpaired *t*-test. Error bars are s.e.m. The boxes in **b-e** show the median and interquartile range, and the whiskers show the minimum and maximum. Statistical significance in **b-e** was determined by pre-set analytic methods of the online tool

The data suggest that MATR3 functions as a putative tumor suppressor and next we scrutinized whether *MATR3* expression has clinical relevance. We first compared *MATR3* expression between normal breast tissues and primary breast tumors. In The Cancer Genome Atlas (TCGA) datasets, we found that *MATR3* expression was higher in the normal breast tissues than in the breast tumors (Fig. 6a). To examine whether *MATR3* expression is correlated with the aggressiveness and poor prognosis of breast cancer, we compared its expression between triple-negative breast cancer (TNBC) and BLBC and other breast cancer subtypes. TNBC and BLBC represent ~ 15–20% of all breast cancer cases and are generally considered highly aggressive and metastatic [24, 25]. RNA-sequencing dataset analysis [26, 27] demonstrated that *MATR3* expression was significantly lower in the TNBC & BLBC compared to the other subtypes (Fig. 6b), which implies that *MATR3* expression is inversely correlated with aggressive and metastatic nature of breast cancer. Furthermore, hormone receptor (HR)-positive breast cancer is known to be less aggressive and correlated with a better prognosis than HR-negative breast cancer, and breast cancer with amplification or high expression of human epidermal growth factor receptor 2 (*HER2 *+) is correlated with a poorer prognosis than *HER2*- breast cancer [25, 28–30]. Interestingly, *MATR3* expression was higher in estrogen receptor (ER)-positive, progesterone receptor (PR)-positive and *HER2*-negative breast cancers than in *ER*-, *PR*-, and *HER2 *+ breast cancers (Fig. 6c–e), which consistently suggests that *MATR3* expression is inversely correlated with aggressiveness and poor prognosis of breast cancer.

**Fig. 7:**
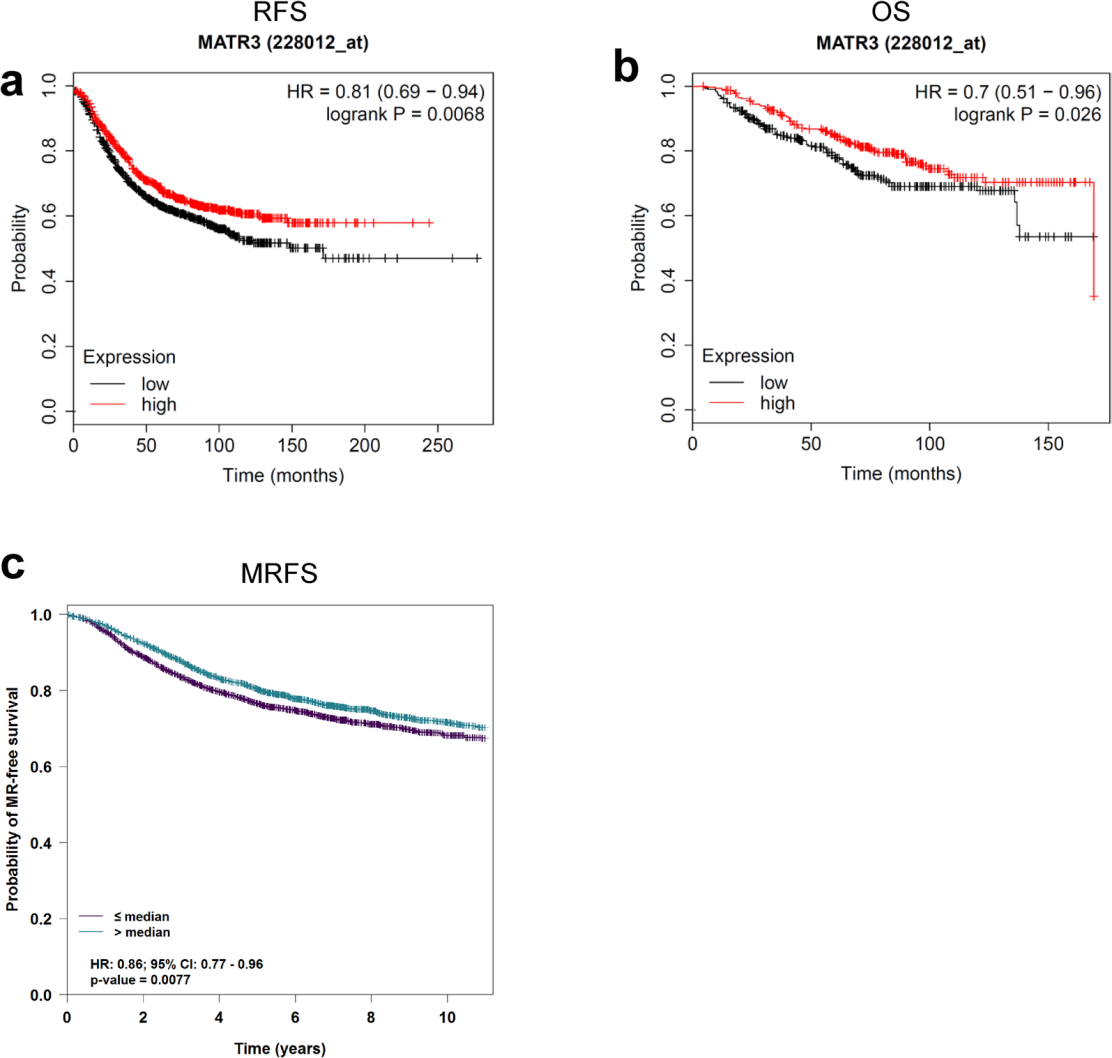
*MATR3* expression is associated with clinical outcomes in breast cancer patients. Kaplan–Meier plots demonstrate that high *MATR3* mRNA levels are associated with better relapse-free (**a**), overall (**b**), and metastatic relapse-free survival (**c**) of breast cancer patients. Patients were stratified by *MATR3* mRNA expression levels (low or high) analyzed from the microarray datasets. *n* = 889 (low in RFS), 875 (high in RFS), 314 (low in OS), 312 (high in OS), 2444 (low in MRFS) and 2395 (high in MRFS). **a**, **b** were plotted at http://kmplot.com/ and **c** was plotted at bc-GenExMiner. Statistical significance was determined by a log-rank test. HR and CI are the hazard ratio and confidence intervals, respectively

We then analyzed how *MATR3* expression is associated with clinical outcomes. By analyzing breast cancer patient survivals with Kaplan–Meier plots, we found that high expression levels of *MATR3* are associated with better patient relapse-free survival (RFS) (Fig. 7a), overall survival (OS) (Fig. 7b)  and metastatic relapse-free survival (MRFS) (Fig. 7c), which strongly suggests that high *MATR3* expression in breast cancer could potentially be a prognostic biomarker to predict better breast cancer patient survival.

## Discussion

So far, MATR3’s role has been relatively well studied in Amyotrophic lateral sclerosis (ALS), a rare human genetic disorder. Especially, heterozygous mutations in *MATR3* are known to cause ALS type 21 (ALS21), which is characterized by vocal cord weakness and pharyngeal dysfunction with distal myopathy [7, 31–34]. However, MATR3’s role in human cancer remains elusive. In cancer, *MATR3* was identified as one of the five genes deleted from chromosome 5 of primary, metastasized and xenografted human basal-like breast cancer (BLBC) [11], which suggests that MATR3 may function as a tumor suppressor gene. In the present study, we demonstrated the potential tumor-suppressive functions of MATR3 in basal-like breast cancer (BLBC). MATR3 suppressed in vitro tumorigenecity, as gauged by cell proliferation and soft agar colony formation assays, promoted apoptotic cell death, as gauged by flow cytometric analyses and PARP cleavage, and migration and invasion, as demonstrated by the changes in EMT markers and in vitro migration and invasion assays. We initially attempted to generate stable MATR3-overexpressing cell lines with various expression vectors to conduct the long-term assays such as soft agar colony formation, but all failed. This may also suggest that forced overexpression of MATR3 in the MATR3-low BLBC cells such as MDA-MB-231 may cause apoptotic cell death, which possibly makes generating stable MATR3-overexpressing cell lines unsuccessful. In the oral squamous cell carcinoma and normal endothelial cells, however, silencing MATR3 using siRNA promoted cell death [35, 36], which indicates that the pro-apoptotic function of MATR3 may be cell-context dependent.

Basal-like breast cancer is an aggressive and metastatic subtype but does not respond to the targeted hormonal therapy due to a lack of hormone receptors such as ER and PR, which demands urgent development of alternative therapeutic strategies. Moreover, immunotherapy including immune checkpoint blockade has provided the cancer research community with the promising anti-tumor effect but advanced BLBC/TNBC is shown to very modestly respond to this therapy [37]. In this sense, MATR3 with potential tumor-suppressive function could serve as a biomarker to predict the efficacy of chemotherapy that promotes BLBC cellular apoptosis; BLBC with high *MATR3* levels may enhance the efficacy of chemotherapeutic agents by promoting apoptosis.

In our initial test shown in Fig. 1a, a small panel of BLBC/TNBC cell lines displays differential MATR3 expression levels. Although genomic deletions or mutations could affect MATR3 gene expression, such alterations are relatively rare in breast cancer according to the mutation analysis (1% or lower, cbioportal.org). However, MATR3 expression levels are known to be regulated post-translationally by being extensively digested by caspases [38] or by being modified through phosphorylation, sumoylation, acetylation, and ubiquitination [39, 40]. Therefore, one possibility is that different post-translational regulation states in each cell line may decide the differential expression of MATR3 although they are all BLBC/TNBC cell lines. The differential MATR3 levels may confer different sensitivity to the apoptosis-inducers such as chemotherapeutic drugs, different degrees of EMT, and ultimately different tumorigenic/metastatic potential, which warrants further investigation.

We conducted bioinformatic analyses to examine the correlation between *MATR3* expression and the aggressive phenotype of breast cancer subtypes. Surprisingly, *MATR3* expression was significantly lower in the aggressive and metastatic breast cancer subtypes and higher expression levels were associated with better breast cancer patient survival, which suggests MATR3’s role as a prognostic biomarker.

MATR3 is an abundant nuclear protein with DNA- and RNA-binding domains, which allows MATR3 to play roles in RNA splicing [10, 41] and gene transcription [6, 42, 43]. Therefore, upon upstream apoptotic cues administered by the chemotherapeutic drugs, MATR3 is activated, which, in turn, may regulate apoptotic gene expression at the RNA levels or control the activity of the apoptosis-related proteins as shown in Table 1 through the interaction. Also, we found that oncogenic YAP/TAZ pathway is the most significantly altered by MATR3 from pathway analysis, and validated that YAP/TAZ target genes were suppressed by MATR3, which suggests that MATR3 functions as a tumor suppressor potentially by regulating YAP/TAZ pathway. Those possibilities and molecular mechanisms also warrant further investigation.

## Conclusions

Here, we demonstrated that MATR3 suppresses tumorigenecity, induces apoptotic cell death, and inhibits migration and invasion of the basal-like breast cancer cells. Our data strongly suggest that MATR3 also offers the possibility to predict aggressiveness and metastatic potential of the given breast cancer and patient survival as a novel cancer prognostic biomarker.

## Methods

### Cell culture

Basal-like/triple-negative breast cancer cell lines, MDA-MB-157, MDA-MB-468, MDA-MB-231, MDA-MB-436, Hs578t, and BT549 were obtained from American Type Culture Collection (ATCC). Cell lines were cultured in the Dulbecco’s Modified Eagle Medium (DMEM) supplemented with 10% fetal bovine serum at the humidified incubator with 5% CO_2_ at 37 °C.

### Plasmids and cloning

BamH1 at 5’ and XhoI at 3’ were chosen to clone ORF of *MATR3* into pCMV-Tag2B (Stratagene) vector. PCR-amplified ORF of *MATR3* sequence was inserted into TOPO vector (Thermofisher, 450,245) and subsequently subcloned into pCMV-Tag2B using two restriction enzyme sites (BamH1 and XhoI). *MATR3* was also cloned into SFB vector [44] by the Gateway cloning method to conduct Tandem affinity purification followed by mass spectrometry (TAP-MS).

### Lentivirus production and infection

Short hairpin RNA (shRNA) clones for *MATR3* in pGIPZ vector were obtained from Horizon Discovery. V2LHS_22224 (sh-MATR3 #1), V3LHS_342293 (sh-MATR3 #2) and V2LMM_10475 (sh-MATR3 #3) clones along with non-silencing control vector were used to produce lentivirus to infect the target cells (BT549). shRNA clones or non-silencing control vector was co-transfected with pCMV-dR8.2 (a gift from Bob Weinberg (Addgene plasmid #8455) and pCMV-VSV-G envelop vector (a gift from Bob Weinberg (Addgene plasmid #8454) into HEK293T cells. 2 days post-transfection, virus supernatant was collected, filtered with 0.45 μm syringe filters and added to the target cells to infect them with 8 μg/ml polybrene. 1 μg/ml puromycin was added to select positive clones.

### Electroporation

MDA-MB-231 cells were subjected to electroporation to introduce pCMV-Tag2B or pCMV-Tag2B-MATR3 plasmids. 4D-Nucleofector (Lonza) with CH-125 program and solution SE was used.

### Total RNA isolation, cDNA synthesis, and real-time quantitative PCR analysis

The samples were prepared as previously described [45]. Briefly, total RNA was isolated from each cell line homogenized in Trizol reagent (Thermofisher) using RNeasy Mini Kit (Qiagen) according to the manufacturer’s instruction. RNA was reverse-transcribed to synthesize cDNA using iScript cDNA Synthesis kit (Bio-rad). Real-time quantitative PCR was performed on a CFX instrument (Bio-rad) with SYBR Green reagent (Bio-rad) and specific primers as followed: *KRT18*-forward, 5′-CAGAGACTGGAGCCATTACTTC-3′; *KRT18*-reverse, 5′-GCCAGCTCTGTCTCATACTTG-3′; *CDH1*-forward, 5′-CTGCCAATCCCGATGAAATTG-3′; *CDH1*-reverse, 5′-TCCTTCATAGTCAAACACGAGC-3′; *CDH2*-forward, 5′-CCCAAGACAAAGAGACCCAG-3′; *CDH2*-reverse, 5′-GCCACTGTGCTTACTGAATTG-3′; *VIM*-forward, 5′-ACCCTGCAATCTTTCAGACAG-3′; *VIM*-reverse, 5′-GATTCCACTTTGCGTTCAAGG-3′; *FN1*-forward, 5′-GTGGCAGAAGGAATATCTCGG-3′; *FN1*-reverse, 5′-GAGAATACTGGTTGTAGGACTGG-3′; *CTGF*-forward, 5′-CGACTGGAAGACACGTTTGG-3′; *CTGF*-reverse, 5′-AGGCTTGGAGATTTTGGGAG-3′; *CYR61*-forward, 5′-GAGTGGGTCTGTGACGAGGAT-3′; *CYR61*-reverse, 5′-GGTTGTATAGGATGCGAGGCT-3′; *AXL*-forward, 5′-TTTATGACTATCTGCGCCAGG-3′; *AXL*-reverse 5′-TGTGTTCTCCAAATCTTCCCG-3′; *AMOTL2*-forward 5′-GACTTCAACCGGGATCTTAGAG-3′; *AMOTL2*-reverse 5′-CCAGCTTCTCTTGCTCCTG-3′; 18 s RNA-forward, 5′-GTAACCCGTTGAACCCCATT-3′; 18 s RNA-reverse, 5′-CCATCCAATCGGTAGTAGCG-3′.

### Western blot analysis

Western blotting was performed as described previously [46]. Antibodies used are as followed: anti-MATR3 (Bethyl Laboratories, #A300-591A), anti-Cyclophilin B (Thermofisher, #PA1-027A), anti-HSP90 (BD Bioscience, #610419), and anti-cleaved PARP (Cell signaling technology, #9541). Expression levels were quantitated based on the density of the blotted bands by normalizing with loading controls (HSP90 or Cyclophilin B) using ImageJ software (https://imagej.nih.gov/ij/).

### In vitro cell proliferation assay

1000 and 1500 of MDA-MB-231 and BT549 cells, respectively, were plated on four 96-well plates with 4-well replicates. Next day (day 1), one plate was taken from the tissue culture incubator, each well was washed once with phosphate-buffered saline (PBS) and the cells were stained with 0.2% crystal violet dissolved in 10% formalin for 10 min. After removing the staining solution, each well was washed with tap water and was air-dried. This procedure was repeated until day 4. Cell stain was extracted with 50 μL of 10% acetic acid and optical density was read at 570 nm using a spectrophotometer.

### Soft agar assay

Soft agar assays to determine in vitro tumorigenecity were performed as described [46]. 1% and 0.7% soft agar (BD Bioscience, #214220) were prepared and mixed 1:1 with 2X supplemented DMEM to make 0.5% bottom agar, and 0.35% top agar containing 2000 cells, respectively, and plated on the 6-well plates.

### Flow cytometry

For cell cycle analysis with propidium iodide, cells were harvested by trypsinizing the adherent cells and washed with phosphate-buffered saline (PBS). The cells were fixed with 100% ice-cold ethanol and washed with PBS after they were pelleted. Stain solution (0.1% TritonX-100, 20 μg/ml propidium iodide (Thermofisher, # BMS500PI) and 200 μg/ml DNase -free RNase A (Thermofisher, #EN0531) in PBS) were added to the cell pellets with the cell pellets vortexed. Resuspended cells were filtered through the cell strainer and subjected to the flow cytometry.

For apoptotic cell death analysis by staining with Annexin V (BD Pharmingen, #550475) and 7-AAD (BD Pharmingen, #51-68981E), cells were treated with serum-free and growth factor-free medium or Staurosporine (Cell Signaling Technology, #9953) at 0.5 μM for 24 h to induce apoptotic cell death. Cells were harvested by trypsinization and washed with PBS. Then cells were stained with APC-labeled Annexin V according to the manufacturer’s protocol and with 7-AAD right before flow cytometric assays. Early (Annexin V +/7-AAD-) and late (Annexin V +/7-AAD +) apoptotic cells were quantitated to determine apoptotic cell death.

### Staurosporine-induced apoptosis

To induce apoptotic cell death, Staurosporine (Cell Signaling Technology, #9953) was added to the cells at the final concentration of 0.5 μM for 24 h. Then cells were harvested for western blotting.

### Migration and invasion assays

In vitro 3-dimensional migration and invasion assays were performed as described [46]. 30,000 cells 60,000 cells resuspended in the serum-free, growth factor-free medium were added to the upper chamber of Boyden Chambers, and serum-containing growth medium was added to the bottom chamber. After 24 h in the humidified incubator, cells on the upper surface of the membrane were removed with a cotton swab and cells on the lower surface of the membrane were stained with 0.2% crystal violet dissolved in 10% formalin. Migrated/invaded cells were examined and counted at 100X magnification.

### Tandem affinity purification followed by mass spectrometry (TAP-MS)

TAP-MS was performed as described [47]. Briefly, SFB-tagged MATR3 was overexpressed in HEK293T cells by direct transfection, and cells were lysed in NETN buffer (20 mM Tris–Hcl at pH 8.0, 100 mM NaCl, 1 mM EDTA, 0.5% Nonidet P-40). MATR3 was serially pulled down with streptavidin Sepharose and S-protein beads. The immunocomplexes were eluted and separated by SDS-PAGE. Mass spectrometry was performed at Taplin Biological Mass Spectrometry Facility of Harvard Medical School.

### Bioinformatic analysis

To identify the most enriched pathway regulated by MATR3, differentially expressed genesets in MATR3-silenced U2OS cells were downloaded from the previous study [9] and analyzed in Enrichr, an online gene list enrichment analysis tool [18]. Enriched pathways altered by MATR3 were retrieved from WikiPathways 2019 (Human) library and each pathway was listed based on their statistical significance (*P* value).

Breast cancer RNA-sequencing datasets (BRCA) of The Cancer Genome Atlas (TCGA) were retrieved from https://xenabrowser.net/ (N = 1218) and *MATR3* expression levels were compared between primary breast tumors and normal breast tissues. *MATR3* expression levels in RNA-sequencing datasets between two different subtypes of breast cancer were compared at http://bcgenex.centregauducheau.fr/ [26, 27] and displayed by Box and Whisker plots. Patient survival (relapse-free, overall, and metastatic relapse-free survival) was plotted by Kaplan–Meier Plots. RFS and OS were plotted at http://kmplot.com/ using Jetset best probe (228012_at) [48] and MRFS were plotted at http://bcgenex.centregauducheau.fr/. Patients were split by median expression and the statistical analyses were performed by pre-set analytic methods.

### Statistical analysis

Data are presented as mean ± s.e.m., and a two-tailed unpaired *t* test was used to compare two groups of independent samples. Statistical analyses using the online bioinformatics tool were performed according to the pre-set analytic methods of each online tool. The log-rank test was used to compare Kaplan–Meier survival curves. *P* < 0.05 was considered statistically significant.

## Data Availability

The TCGA dataset is available and can be downloaded free at https://xenabrowser.net/datapages/.

## Abbreviations

BLBC: Basal-like breast cancer
TNBC: Triple-negative breast cancer
MATR3: Matrin 3
PARP: Poly (ADP-ribose) polymerase
EMT: Epithelial-mesenchymal transition
PCR: polymerase chain reaction
HER2: Human epidermal growth factor receptor 2
ALS: Amyotrophic lateral sclerosis
TAP-MS: Tandem affinity purification followed by mass spectrometry
KRT18: Cytokeratin 18
CDH1: E-cadherin
CDH2: N-cadherin
VIM: Vimentin
FN1: Fibronectin
AMOTL2: Angiomotin Like 2
CTGF: Connective Tissue Growth Factor
CYR61: Cysteine Rich Angiogenic Inducer 61
TCGA: The Cancer Genome Atlas
HR: Hormone receptor
ER: Estrogen receptor
PR: Progesterone receptor
RFS: Relapse-free survival
OS: Overall survival
MRFS: Metastatic relapse-free survival
shRNA: Short hairpin RNA
siRNA: Small interfering RNA
